# Extracellular Vesicle Integrins Distinguish Unique Cancers

**DOI:** 10.3390/proteomes7020014

**Published:** 2019-04-11

**Authors:** Stephanie N. Hurwitz, David G. Meckes

**Affiliations:** Department of Biomedical Sciences, Florida State University College of Medicine, Tallahassee, FL 32306, USA; snh13d@med.fsu.edu

**Keywords:** Exosomes, mass spectrometry, proteomics, biomarkers, cancer, extracellular vesicles, microvesicles, oncosomes

## Abstract

The proteomic profile of extracellular vesicles (EVs) has been of increasing interest, particularly in understanding cancer growth, drug resistance, and metastatic behavior. Emerging data suggest that cancer-derived EVs carry an array of oncogenic cargo, including certain integrin proteins that may, in turn, promote cell detachment, migration, and selection of future metastatic sites. We previously reported a large comparison of secreted vesicle protein cargo across sixty diverse human cancer cell lines. Here, we analyze the distinct integrin profiles of these cancer EVs. We further demonstrate the enrichment of integrin receptors in cancer EVs compared to vesicles secreted from benign epithelial cells. The total EV integrin levels, including the quantity of integrins α6, αv, and β1 correlate with tumor stage across a variety of epithelial cancer cells. In particular, integrin α6 also largely reflects breast and ovarian progenitor cell expression, highlighting the utility of this integrin protein as a potential circulating biomarker of certain primary tumors. This study provides preliminary evidence of the value of vesicle-associated integrin proteins in detecting the presence of cancer cells and prediction of tumor stage. Differential expression of integrins across cancer cells and selective packaging of integrins into EVs may contribute to further understanding the development and progression of tumor growth and metastasis across a variety of cancer types.

## 1. Introduction

Integrins are well-conserved ubiquitous cell adhesion receptors that play important roles in extracellular matrix attachment and signal transduction, contributing to pathways involved in cell growth, survival, proliferation, and migration. Functional integrin molecules are heterodimers composed of two integral membrane glycoprotein subunits, one α and one β, held together by disulfide bonds. Serving as an extracellular matrix linker, the globular head of an integrin protein projects extracellularly, while the C-terminal tails of both subunits anchor actin cytoskeleton components inside the cell [1,2,3,4,5,6,7,8,9]. In vertebrates, the integrin family comprises 18 α subunits and eight β subunits that can assemble into 24 heterodimers with various ligand-binding properties [3]. While a variety of ligands bind integrin receptors, non-collagenous matrix proteins containing the arginine-glycine-aspartate (RGD) sequence, including fibronectin, laminin, and vitronectin serve as major extracellular ligands for integrin molecules [4,10]. The majority of integrin cytoplasmic tails bind anchor proteins such as talin, α-actinin, and filamin which connect bundles of actin filaments. The ability of integrins to bind both intra- and extra-cellular proteins facilitates the transmission of force across the plasma membrane, and also contributes to their unique bidirectional signaling properties [11,12,13,14,15]. “Outside-in” integrin signaling describes the sequence of extracellular ligand binding and the subsequent activation of intracellular cytoskeletal components as well as various enzymes, including the focal adhesion kinase (FAK), Src-family kinases, small GTPases Ras and Rho, ABL-family kinases, and integrin-linked kinase (ILK) [16]. The mitogen-activated protein kinase (MAPK)/extracellular signal-regulated kinase (ERK), and the c-Jun N-terminal kinase (JNK) pathways are activated through these transduction mechanisms, providing integrin-dependent regulation of cell cycle and proliferation [17]. The FAK-Src complex also facilitates cell migration by disassembling focal adhesion complexes at the trailing edge of the cell [18]. Furthermore, “inside-out” signaling occurs following cytokine or chemokine activation of G protein-coupled receptors (GPCRs) which leads to integrin activation, assembly, and increased extracellular ligand affinity [3,19].

The apparent multifaceted role of integrins in cell growth and migration has generated great interest in the contribution of this protein family to cancer progression. Accumulating evidence suggests integrin signaling plays multiple roles in the stepwise progression of cancer, including tumor growth, detachment, angiogenesis, intravasation, homing and extravasation, and finally the expansion of metastases [20,21,22,23,24,25,26,27,28,29]. It is believed that these mechanisms of cancer progression may be, in part, regulated by integrin-switching. In these scenarios, integrins that promote cell adherence and quiescence are downregulated, while those that foster the breakdown of cell adhesions and remodeling of the extracellular matrix (ECM) may be simultaneously overexpressed [20,30].

It has been well understood that cellular integrin trafficking occurs through constitutive caveolin- or clathrin-mediated endocytosis into endosomes for sorting, degradation, or recycling [31]. Integrin localization to endosomes may also be important for FAK signaling [32]. More recently, integrin secretion into small 40–200 nm extracellular vesicles (EVs), including exosomes and small microvesicles, secreted from cells has been described [33,34,35,36,37,38,39,40,41]. The roles of extracellular vesicles in multiple steps of cancer progression have also been of great interest [42]. In a seminal study published by Hoshino and colleagues in 2015, tumor-derived EVs were demonstrated to harbor integrins that were instrumental in preparing a pre-metastatic tumor niche and indeed guided organ-specific metastasis based on EV-cell tropism [38]. Vesicles containing integrins α6β1 and α6β4 were found to be taken up specifically by lung fibroblasts and epithelial cells and were associated with lung metastasis of progenitor cancer cells. In contrast, vesicle αvβ5 directed uptake of EVs by liver Kupffer cells and promoted liver metastasis. Small peptide inhibition of these respective integrin receptors significantly reduced cancer metastasis to distant sites [38]. In another study, prostate cancer EVs were shown to transfer integrin αvβ6 to non-tumorigenic cells, resulting in an increased migratory capacity of the recipient cells [37]. Similarly, EVs carrying αvβ3 from prostate cancer cells have been observed to increase cell migration of surrounding recipient cells [35,36]. In the context of viral tumorigenesis, increased levels of integrin subunits α4, αL, and β3 were demonstrated to be secreted into EVs following infection with the human tumor virus Epstein-Barr virus (EBV); while α3, α6, and β1 integrins were decreased in EVs from EBV or Kaposi sarcoma herpesvirus infected cells [39]. Finally, persistent EV secretion of integrin subunits, such as β4 in the setting of prostate carcinoma, may also be useful in predicting chemotherapy-resistant cells [34].

Altogether, a growing body of evidence suggests that integrins secreted into cancer EVs may confer invasive or migratory phenotypes to naïve surrounding cells, and can direct organotropic metastasis by site-specific uptake and microenvironment modification. Furthermore, EV integrins offer novel targets as biomarkers for cancer progression. In a previous study, we conducted a large-scale proteomic analysis of extracellular vesicle cargo secreted from a panel of sixty human cancer cells [40]. Here we further analyze the EV integrin profiles from these cancer cells. We additionally compare the EV proteomes of breast cancer cells to that of a benign breast epithelial cell line, highlighting the overexpression of many integrin subunits secreted from tumorigenic cells. Finally, the levels of several vesicle-associated integrins correlate with increasing tumor stage and reflect cellular levels. These findings support the utility of circulating integrins as potential cancer cell biomarkers and emphasize the functional roles these proteins play in stepwise cancer progression.

## 2. Materials and Methods

### 2.1. Cell Culture

Sixty cell lines from the National Cancer Institute (NCI-60) were acquired and cultured, as previously described [40]. MCF10a cells were grown using the Mammary Epithelial Cell Growth Medium BulletKit (Lonza, Basel, Switzerland, CC-3150) comprised of the basal medium MEBM supplemented with the provided aliquots of bovine pituitary extract (BPE), human recombinant epidermal growth factor (hEGF), insulin, and hydrocortisone. Instead of the GA-1000 aliquot provided in the kit, 100 ng/mL of cholera toxin was added to the medium, as recommended by the American Type Culture Collection (ATCC). At 90% confluence, the complete medium was aspirated and cells were washed with warm sterile phosphate buffered saline (PBS). To minimize contaminating proteins, cells were grown in BPE-free medium for another 48 h before EV enrichment.

### 2.2. Extracellular Vesicle Enrichment and Protein Quantification

NCI-60 and MCF10a EVs were processed as previously described in great detail [40,43]. The enrichment efficacy and purity of samples has been demonstrated numerous times through nanoparticle tracking, immunoblot analysis, and electron microscopy by our laboratory [43,44,45,46,47]. Briefly, serum-free cell-conditioned medium was aspirated from cell culture plates in three biological replicates, and centrifuged at 500× *g* for 5 min, then at 2000× *g* for 30 min before incubation overnight with a 1:1 volume of 2X PEG solution (16% (*w*/*v*) polyethylene glycol, 1 M NaCl). Following the polyethylene glycol incubation, samples were centrifuged at 3214× *g* for 60 min, and pellets were resuspended in PBS for an ultracentrifugation purification step (100,000× *g* for 70 min). Final pellets were lysed in strong lysis buffer (5% SDS, 10 mM EDTA, 120 mM Tris-HCl pH 6.8, 2.5% β-mercaptoethanol, 8 M urea) with the Halt^tm^ protease inhibitor (ThermoFisher, Waltham, MA, USA, 78438) added. Vesicle protein quantification was performed using the fluorescence-based EZQ™ Kit (ThermoFisher, Waltham, MA, USA, R33200) according to the manufacturer’s instructions.

### 2.3. SDS-PAGE and In-Gel Digestion

NCI-60 EV protein was further processed as previously reported [40]. Similarly, in this study, 20 µg of MCF10a EV protein was loaded into a 4–20% polyacrylamide gel (Lonza, Basel, Switzerland, 59511) for sodium dodecyl sulfate polyacrylamide gel electrophoresis (SDS-PAGE), gel fixation, and Coomassie staining as described in detail [48]. Gel lanes were fractionated into five sections and then subdivided into 1 mm^3^ cubes for trypsin digestion [43].

### 2.4. Mass Spectrometry

Mass spectrometry and protein identification of NCI-60 EV cargo were reported in our previous study [40]. Additional analysis of NCI-60 integrin proteins was performed using data published in the Supplementary Table S1 of our prior study. Trypsin-digested MCF10a EV protein samples were submitted to the Florida State University Translational Science Laboratory for liquid chromatography-tandem mass spectrometry (LC-MS/MS) analysis, consistent with the protocol and parameters used previously for NCI-60 cell-derived EVs [40]. The same Thermo LTQ Orbitrap Velos nLC-ESI-LTQ-Orbitrap (high-resolution electrospray tandem mass spectrometer) was used in this study to analyze the MCF10a EV protein. Raw peptide data from each of the five fractions of the MCF10a samples were pooled and analyzed in the Scaffold software. Data was analyzed using three search engine databases (MS-Amanda Proteome Discoverer, Mascot, and Sequest) and a recent UniProt knowledgebase reviewed (Swiss-Prot) human protein database. Fragment tolerance was set to 0.80 Da (monoisotopic). Fixed modifications included only carbamidomethyl (C), and variable modifications included oxidation (M), N-terminal acetylation, and phosphorylation (STY). Digestion mode was set specific for trypsin, with a maximum of two missed cleavages. The false discovery rate was set to 0.01. MCF10a EV data were averaged amongst replicates, then normalized to previously analyzed NCI-60 EV proteins by multiplying the integrin spectral counts by a calculated normalization factor (average total spectral counts per sample across all the benign and cancer breast EVs divided by the total spectral counts identified in each sample). Whole cell protein data published by Gholami et al. [49] was previously normalized across the NCI-60 panel [40], and integrin expression was re-analyzed in this study. Previously published mass spectrometry analysis of EVs from an additional non-tumorigenic prostate epithelial cell line (PNT1A) [50] was utilized to compare integrin levels to various staged tumor-derived samples in Figure 4. Spectral counts of PNT1A EV proteins identified by Worst and colleagues were averaged amongst replicates, then corrected by a normalization factor (average total spectral counts identified in PC3 prostate cancer EVs in the NCI-60 panel divided by the total spectral counts identified in PC3 EVs within the same Worst et al. study [50]). Normalized spectral count values were used in subsequent analyses in this study.

### 2.5. Protein Enrichment Analyses

Differential enrichment of biological processes between proteins identified in the MCF10a EVs and proteins identified in all six breast cancer EV samples was compared using FunRich v3 [51,52]. All terms with an adjusted *p*-value less than 0.5 were determined to be significant. Displayed results reflect the processes containing the highest percentage of proteins from each dataset.

### 2.6. Statistical Analyses

Differential expression of NCI-60 EV integrin proteins was previously analyzed by DESeq2, as described in detail [40]. Simple linear regression analysis was performed to examine correlations between cellular and EV integrin levels in this study. Relationships between levels of alpha and beta subunits secreted into EVs were similarly analyzed by linear regression analysis. Statistical significance of integrin levels across various tumor stages was evaluated by a single factor analysis of variance (ANOVA) with post-hoc pairwise comparisons. Confidence intervals of 95% were used. Figures were constructed using Microsoft Excel and CorelDraw X5 software.

## 3. Results

### 3.1. Extracellular Vesicle Integrin Profiling across the NCI-60 Panel

**Figure 1:**
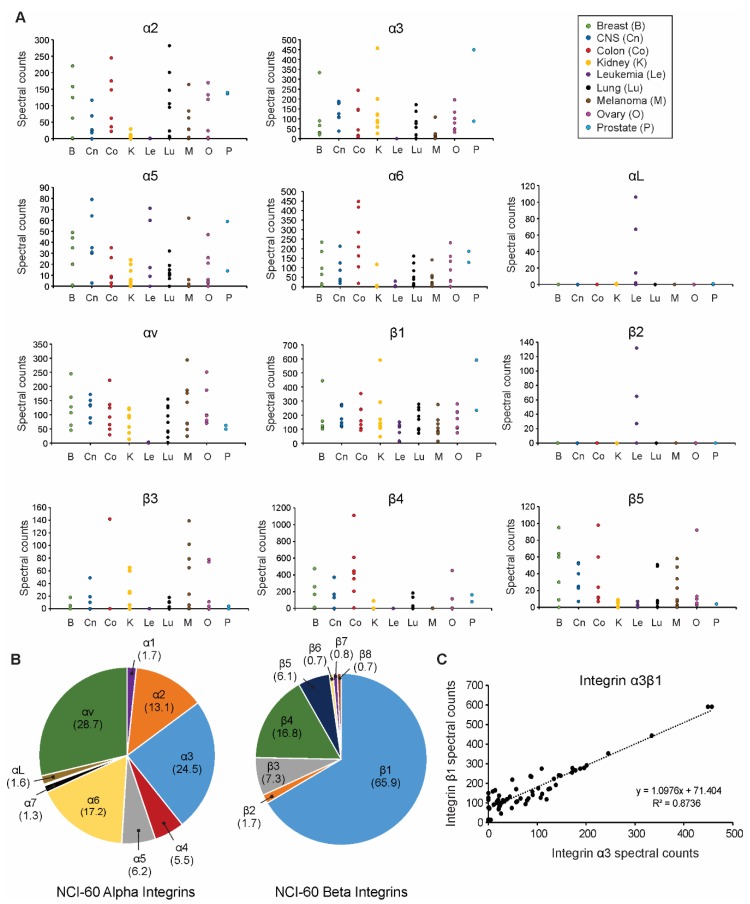
Tumor extracellular vesicles display distinct integrin profiles. (**A**) Spectral counts of alpha and beta integrin subunits secreted into tumor extracellular vesicles (EVs) were compared across the NCI-60 panel of human cancer cells; (**B**) Breakdown of EV alpha and beta integrin subunit composition (percentages) averaged across the NCI-60 panel; (**C**) Direct comparison of vesicular α3 and β1 protein spectral counts secreted by each of the 60 cancer cell lines.

In a prior study, we reported a comprehensive comparison of extracellular vesicle proteins secreted from sixty human cancer cells, highlighting both common and differentially expressed cargo [40]. Here, we closely analyze the integrin profiles of EVs harvested across the panel of cancer cells. A striking number of integrins were found to be significantly differentially expressed across cell-derived EV samples, including alpha integrins α2, α3, α4, α6, α7, and αv, as well as beta integrins β1, β4, β5, and β8. As we previously highlighted [40], integrin β1 was universally present in varying levels within EVs secreted from all cells, while other integrins were more selectively expressed (Figure 1A, Figures S1 and S2). For instance, integrins α2, α3, α6, and αv were generally secreted into EVs from solid tumors. On the other hand, β2 integrin subunits are present exclusively on the surface of leukocytes and were likewise found only to be secreted along with αL into the EVs from leukemia cells in this study. Subunits α2, α3, α6, αv, β1, and β4 comprised the vast majority of integrins secreted into cancer EVs (Figure 1B). Interestingly, integrin β1 has been previously proposed to be more highly expressed in vesicles secreted from cancer cells than non-tumorigenic cells [38]. Because the β1 and β4 subunit encompassed the chief beta subunits identified in cancer EVs, commonly secreted alpha subunits were compared to beta subunit secretion. Spectral counts of integrins α2, α3, α6, and αv were plotted against integrin β1 and β4 spectral counts across the NCI-60 EVs (Figure S3). Integrin α3 was found to be most often secreted in a one-to-one ratio with the β1 subunit (Figure 1C). This positive correlation and ratio highly suggest the abundance of integrin α3β1 secreted into cancer EVs.

### 3.2. Selected Vesicle Integrin Proteins Reflect Progenitor Cell Expression

**Figure 2:**
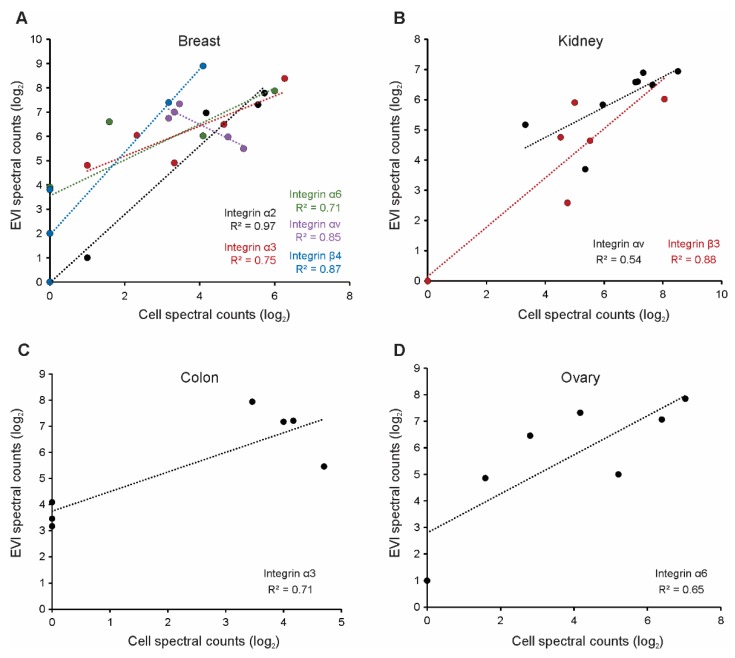
Various vesicle-associated integrins represent progenitor cell expression across epithelial cancer lines. Spectral counts of integrin subunits in (**A**) breast; (**B**) kidney; (**C**) colon; and (**D**) ovarian cancer-derived EVs compared to respective whole cell spectral counts previously reported.

In our previous study, we also reported that many proteins secreted into EVs reflect their progenitor cell levels, suggesting the utility of vesicle cargo as cellular biomarkers [40]. Here, normalized spectral counts of abundant integrin subunits secreted into breast cancer EVs were compared to whole cell levels previously published [49] (Figure 2A). Integrin α2 levels in vesicles were noted to correlate highly with the respective progenitor cell levels, in a nearly 1:1 ratio. Integrins α3, α6, and β4 also demonstrated positive correlations between the cell and vesicle expression, though notably with lower coefficients of determination. Interestingly, integrin αv demonstrated a strongly negative correlation between cell and vesicle protein levels, perhaps suggesting a more selective packaging of the protein into EVs. Correlations between cell and vesicle integrin levels were further examined amongst several other epithelial cell lines. Vesicle integrins αv and β3 were positively correlated with progenitor cell levels in kidney-derived EVs (Figure 2B), while vesicle integrins α3 and α6 best-represented cellular levels in colonic and ovarian tissue, respectively (Figure 2C,D).

### 3.3. Integrin Expression Differs in Cancer Cell-Derived EVs Compared to Benign EVs

**Figure 3:**
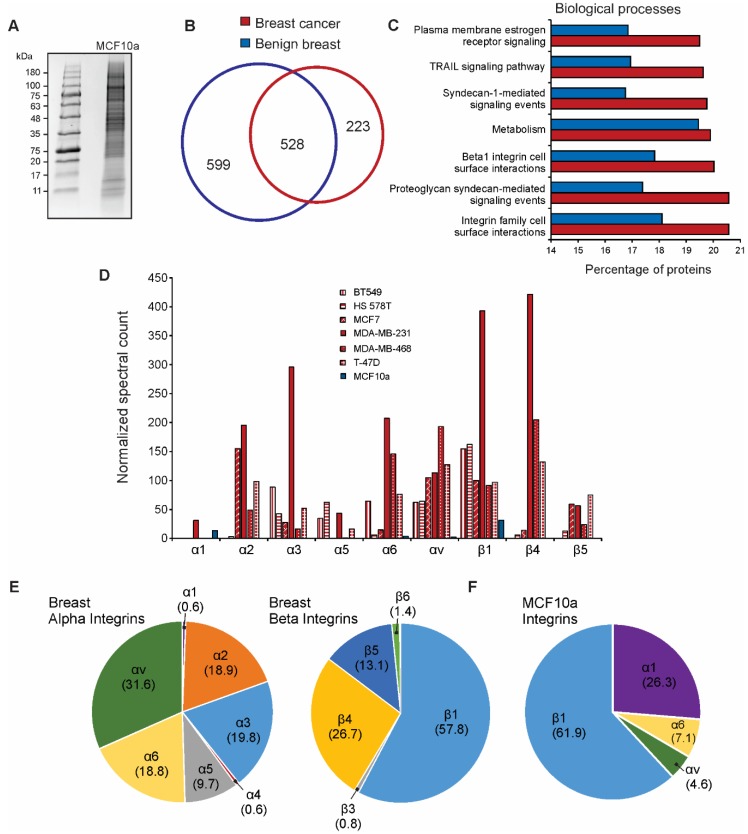
Breast cancer EV integrin profiles differ from benign breast cell-derived vesicles. (**A**) Coomassie-stained gel purification of MCF10a EV proteins; (**B**) Overlap of total vesicle proteins identified by mass spectrometry from MCF10a cells (benign breast) compared to those commonly identified in all six breast cancer cell lines in the NCI-60 panel; (**C**) Enrichment analysis of proteins identified in benign breast EVs versus breast cancer cell-derived EVs; (**D**) Spectral count comparison of the most abundant integrin subunits secreted by benign (blue) or tumor (red) breast cells; (**E**) Breakdown of EV alpha and beta integrin subunit composition (percentage of total alpha or beta proteins, respectively) secreted from breast cancer cells; (**F**) Vesicle composition of integrin subunits secreted by MCF10a breast epithelial cells (percentage of total integrins identified in samples).

Multiple integrins secreted into breast cancer-derived EVs appeared to reflect respective cellular protein levels. To begin to understand potential changes in tumor-derived EVs, we focused on the further examination of the vesicle integrin profiles from breast tissue. Extracellular vesicles from the benign breast epithelial cell line MCF10a were harvested and similarly purified for mass spectrometry analysis (Figure 3A and Table S1). Proteins common to all six breast cancer-derived EVs were compared to those found in MCF10a EVs (Figure 3B). Significant overlap between common cancer and benign EV proteins was seen, although a number of proteins appeared to be present or absent only in breast cancer cell-derived EVs. In an unbiased enrichment analysis, proteins enriched in breast cancer EVs were involved in biological processes including integrin surface interactions, β1 integrin interactions, and syndecan-mediated signaling (Figure 3C). Interestingly, normalized spectral count comparison of integrin subunits across breast cell-derived EVs demonstrated significant overexpression of integrins present in cancer EVs (Figure 3D). Only integrins α1, α6, αv, and β1 were identified in benign breast EVs. Notably, integrin α1 appeared to be present at higher levels in benign breast EVs than most cancer EVs. The breakdown of common alpha and beta integrin subunits found in breast cancer EVs closely resembled the general profile of cancer EV integrins (Figure 1B and Figure 3E). While integrin β1 remained the predominant beta subunit in benign breast EVs, integrin α1 comprised the majority of alpha subunits (Figure 3F). Integrins α2, α3, and α5 were not present in benign EVs compared to cancer EVs. Differences in these expression profiles suggest that “integrin switching” may be reflected in vesicles secreted from progenitor cancer cells.

### 3.4. EV Integrin Levels Predict Cancer Stage

**Figure 4:**
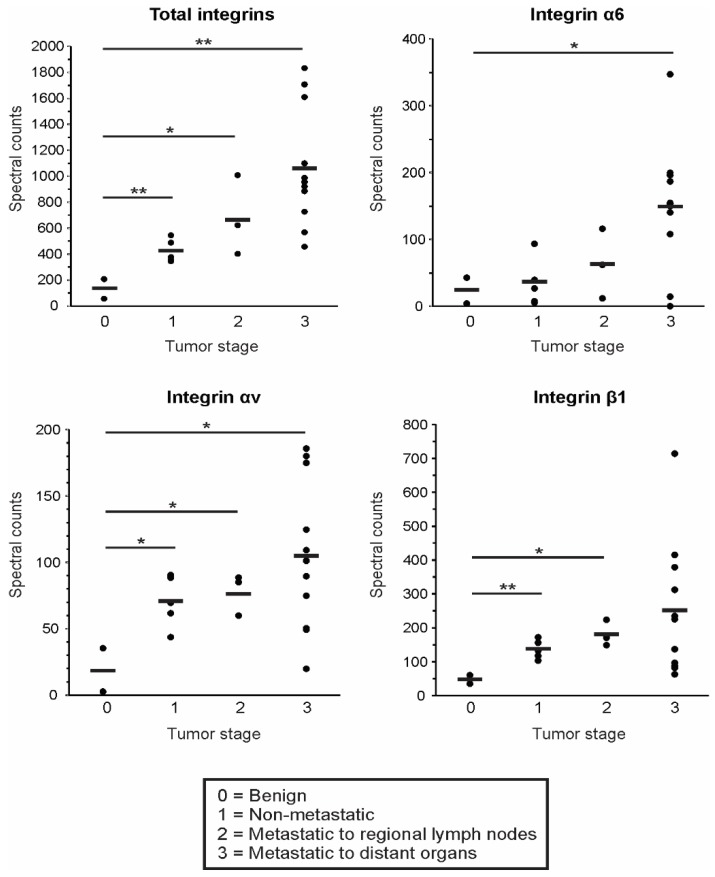
Epithelial cancer stage correlates with EV integrin levels. Spectral counts of representative vesicle integrin subunits compared across benign and tumorigenic cancer lines. Tumor stage was categorized based on clinical information provided by the American Type Culture Collection (ATCC) database. Average spectral counts of integrins within each tumor stage were compared to benign cell-derived EV levels by ANOVA. *p < 0.05, **p < 0.01.

The data in this study support the hypothesis that integrin levels are likely increased in vesicles secreted from tumorigenic breast cells compared to benign breast epithelial cells. To examine EV integrin levels across tumor stages, breast cancer lines were categorized by the relative tumor stage according to clinical information provided by the American Type Culture Collection (ATCC) database. Additional NCI-60 epithelial cancer lines from colon, kidney, lung, ovarian, and prostate tissue with available ATCC clinical information were categorized and examined here as well. As tumor staging varies substantially across specific cancer types and is difficult to directly compare across tissue types, staging criteria in this study was determined as follows: stage 1 denoted non-metastatic cells (HS578T, HT29, 786-O, IGROV1, and OVCAR-3), stage 2 denoted tumors that had spread to regional lymph nodes (BT549, HCT-15, and NCI-H522), and stage 3 included those with documented metastasis to distant organs (MCF7, MDA-MB-231, MDA-MB-468, T47D, Colo205, SW620, CAKI, NCI-H226, SK-OV-3, DU145, and PC3). Tumor-derived EVs were compared to vesicles secreted from benign MCF10a cells, as well as previously published mass spectrometry analysis of benign prostate epithelial cells (PNT1A cells) [50]. Benign MCF10a and PNT1A cells were classified as stage 0. Total spectral counts of all integrin proteins identified in cell-derived EVs were seen to increase in samples originating from advancing stages of the tumor (Figure 4). In particular, higher levels of integrins α6, αv, and β1 were secreted from more aggressive progenitor cancer cells. These preliminary findings demonstrate the potential utility of circulating integrin proteins to detect early tumors and predict more aggressive cancers.

## 4. Discussion

Generating increasing excitement across many scientific fields, the trafficking patterns and content of extracellular vesicles have shed recent light onto mechanisms of cancer growth and metastasis. In this study, we focus on the differential expression and packaging of integrin proteins into EVs from a variety of cancer cell types. While EV integrin content may serve as a unique circulating fingerprint representing an underlying malignancy, several integrin proteins may be similarly packaged into many cancer-derived EVs. Here, we highlight integrin subunit β1 as a commonly secreted integrin into the majority of EVs. Despite prior evidence suggesting the β1 subunit is only secreted in cancer-derived EVs, here we identified this protein secreted in low levels from benign cells as well. However, we demonstrate that in cancer cell-derived EVs, the β1 subunit specifically couples to a high degree with the α3 subunit to form a functional integrin protein heterodimer. Interestingly, the α3β1 integrin has been previously demonstrated to preferentially bind laminins involved in extracellular matrix assembly [53,54] and decreased expression has been noted in many epithelial cancer cells [23,55,56,57,58,59,60]. Interaction of membrane-bound α3β1 integrin with the adjacent extracellular matrix may serve to inhibit cellular invasion through outside-in signal transduction. Indeed, increased expression of several oncoproteins, including n-Myc and c-Myc have been found to be associated with decreased cellular α3β1 integrin levels [61,62,63]. Given that α3β1 integrin is known to interact with several tetraspanin proteins including CD63 and CD81 [64], it is possible that EV secretion of α3β1 integrin may serve to decrease intracellular levels, thereby facilitating downstream oncogenic pathways. We have previously demonstrated CD63-dependent protein secretion into EVs as a major mechanism to regulate intracellular signaling activity in this manner [45,47].

In this study, we also identified the vesicle-associated integrin subunit α2 as highly correlated with breast cell levels, suggesting the utility of this protein as a representative circulating biomarker. The α2 subunit has previously been proposed as a marker of advancing colorectal cancer [65] and has been additionally implicated in prostate cancer metastasis to the bone [66]. Vesicle levels of integrin α6 similarly reflected progenitor cell levels in breast and ovarian tissue, and furthermore was significantly increased in EVs derived from later stage metastatic cells. These data suggest the potential utility of EV-associated α6 protein as a marker of advancing epithelial cancer, and particularly reflective of breast and ovarian cell expression.

Strikingly, total EV integrin levels appeared to be present in accumulating levels as the cancer stage increased. Integrins α6, αv, and β1 were individually observed to significantly increase with tumor stage. While this study provides profiling of EV integrins across a wide variety of progenitor cells, we certainly acknowledge the limitation of using immortalized cell lines rather than primary tumor specimens. While the NCI-60 panel provides the opportunity to analyze a wide variety of cancer cell types from various tissue sources, clinical information available to researchers regarding the original specimens is sparse. Further investigation is clearly warranted to assess the clinical application of these vesicle integrins in the context of cancer diagnosis. Certainly, the future study of the unique EV integrin fingerprints from varying cancer types highlighted in this study may facilitate further application to diagnosing malignancies in clinical settings.

Finally, although beyond the scope of this study, our findings here present an opportunity to further explore the roles of vesicle integrins in cancer metastasis and tissue targeting. As mentioned above, significant work from the Lyden lab has provided evidence that EV integrins may guide tissue-specific metastases into lung or liver microenvironments [38,67]. Here we expand on the proteomic analysis of cancer cell-derived EV integrins, creating a profile across sixty unique cancer cells with a propensity to seed to various metastatic sites. We also compare vesicle integrin expression between benign and tumorigenic epithelial cells, demonstrating increased integrin secretion and integrin-switching in cancer EVs that suggests a role of these vesicle proteins in cancer progression. Altogether, the analyses conducted in this study provide evidence of widespread differences in integrin secretion across various cancer cell types and further suggest changes in cancer EV integrins compared to those secreted from benign cells. Given the growing body of evidence surrounding the roles of integrin proteins in the stepwise progression of cancer and the abundance of integrins present in cancer EVs, recognition and elucidation of the differential integrin secretion from cancer cells may deliver novel means to understand global mechanisms of tumorigenesis and metastasis. Furthermore, future investigations into integrin-directed EV uptake will be essential to facilitating innovative vesicle engineering for targeted or therapeutic delivery of cargo.

## Supplementary Materials

The following are available online at https://www.mdpi.com/2227-7382/7/2/14/s1, Table S1: Proteins identified from mass spectrometry analysis of MCF10a cell-derived EVs; Figure S1: Alpha integrin subunit expression secreted into EVs across the NCI-60 panel of cancer cells; Figure S2: Beta integrin subunit expression secreted into EVs across the NCI-60 panel of cancer cells; Figure S3: The relation of the most abundant alpha and beta integrin ratios secreted into cancer-cell derived vesicles.
