# Supplementary figures and images for: Extracellular Vesicle Integrins Distinguish Unique Cancers
Source: Proteomes. 2019 Apr 11;7(2):14. doi: 10.3390/proteomes7020014 (PMC6630702; doi:10.3390/proteomes7020014)

Supplementary figure 1

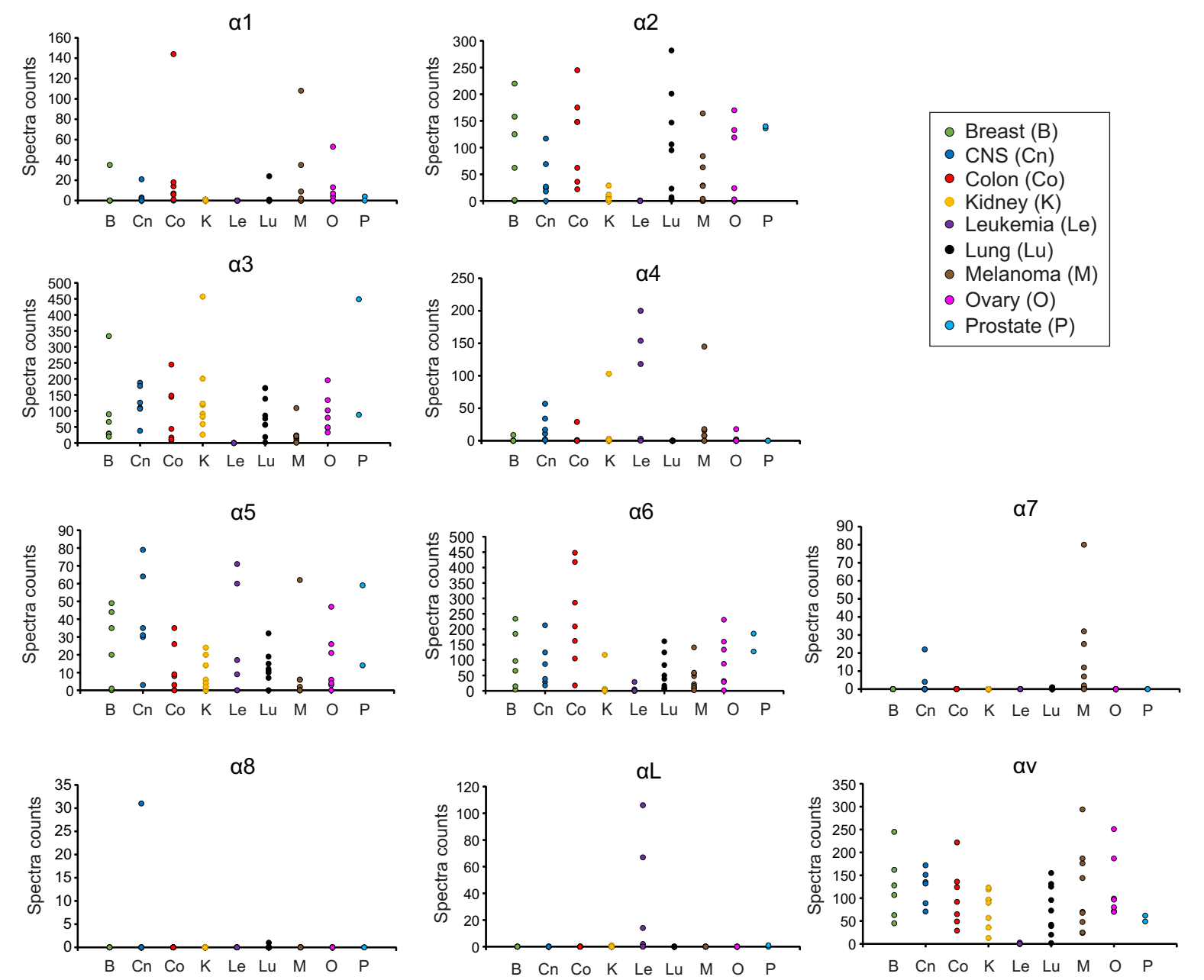

Supplementary figure 2

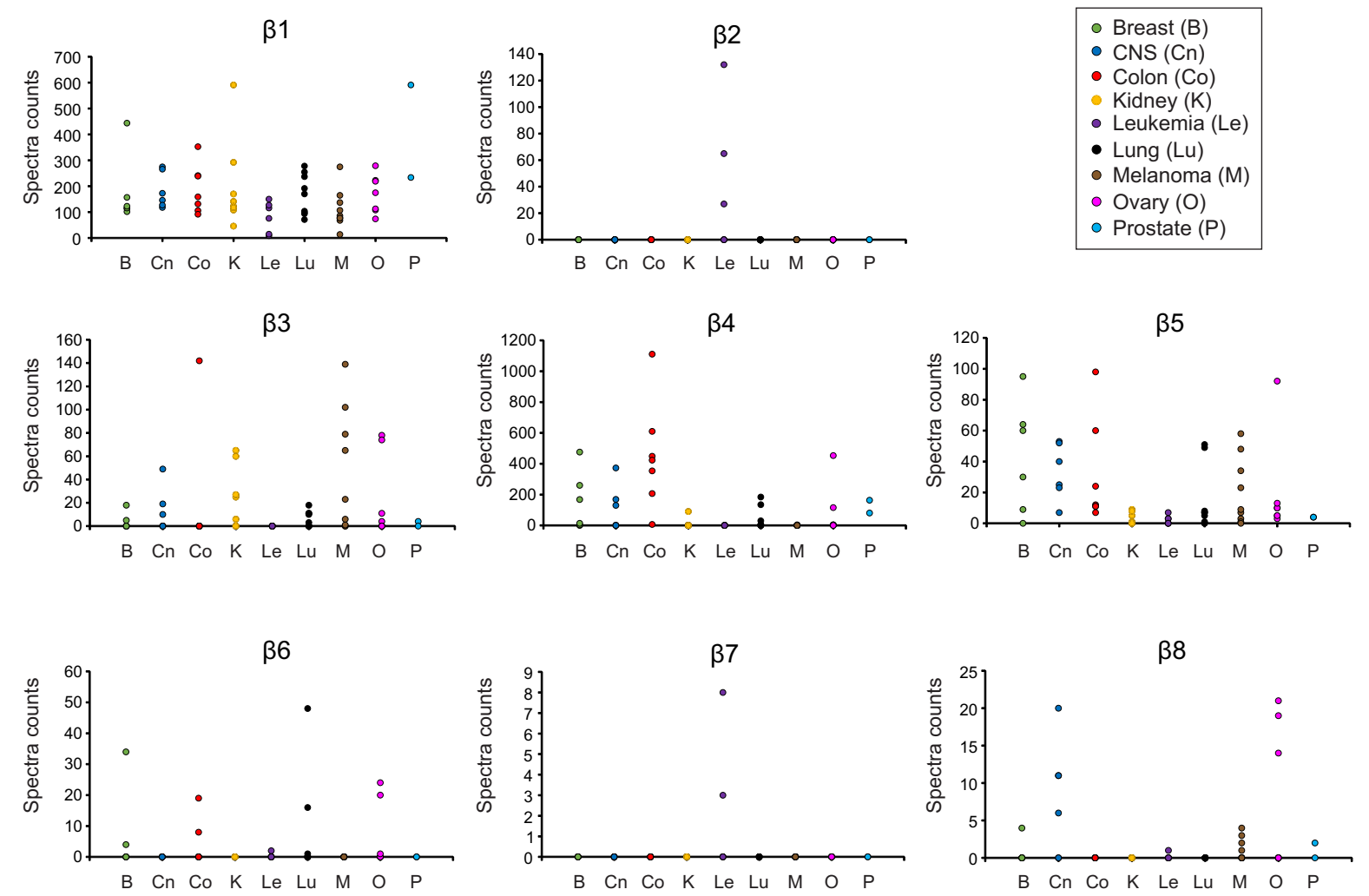

# Supplementary figure 3

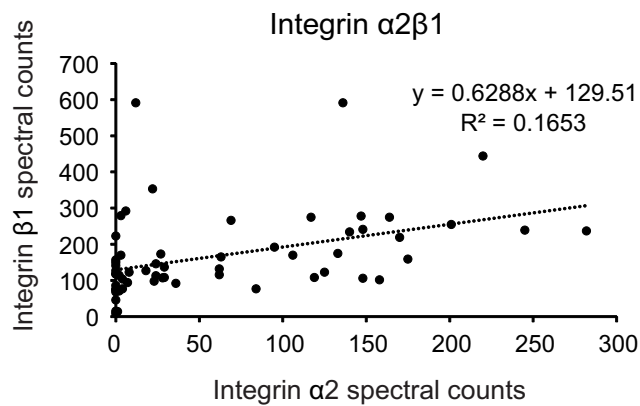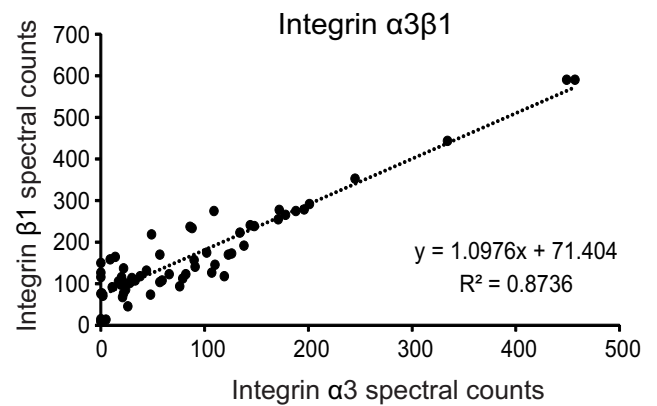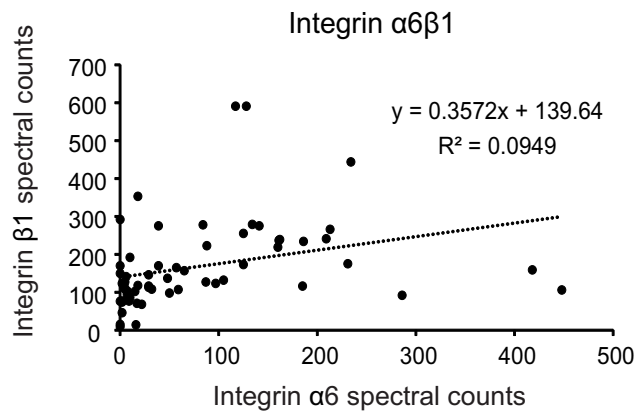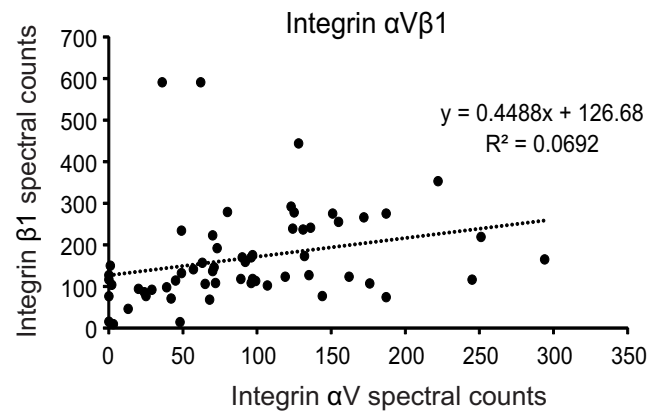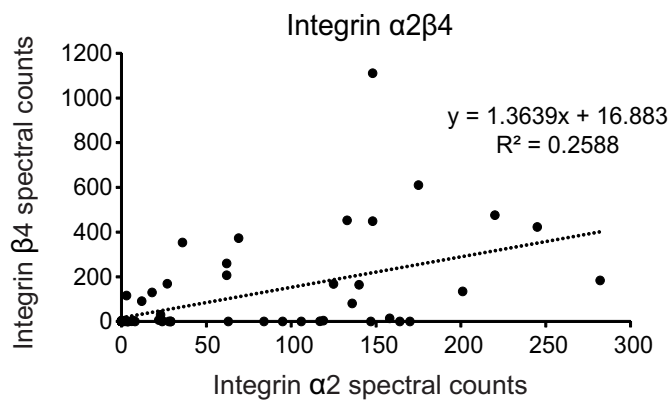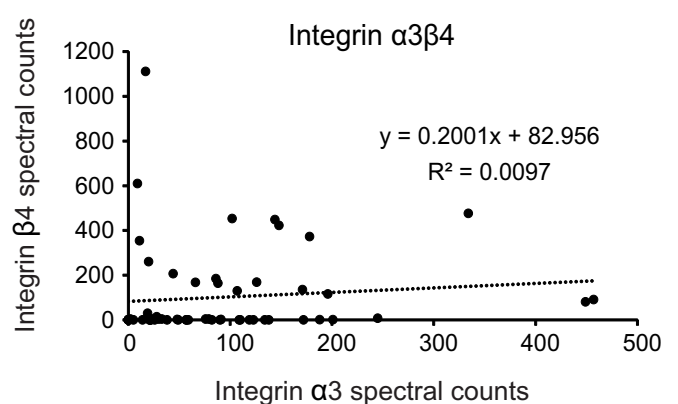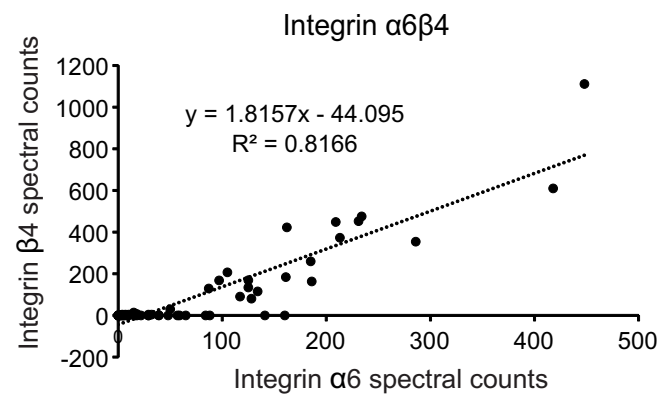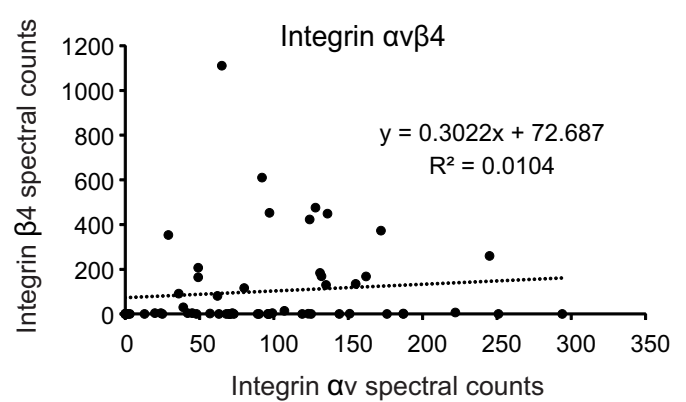

Supplement: Supplementary file 1 [file proteomes-07-00014-s001.zip › supplementary-revised/Supplementary figures_revised.pdf]
